# Spatial architecture of tumor-infiltrating lymphocytes adds predictive value beyond stromal TIL density for neoadjuvant response in triple-negative breast cancer

**DOI:** 10.3389/fimmu.2026.1879640

**Published:** 2026-09-03

**Authors:** Ke Yang, Hua Huang, Wei Shen, Yang Liu, Daxin Gao, Tian Hao, Haiyu Zhou, Yonghong Huang

**Affiliations:** 1 Department of Orthopedics, Chengdu Xinhua Hospital, Chengdu, Sichuan, China; 2 The Second Hospital & Clinical Medical School, Lanzhou University, Lanzhou, Gansu, China; 3 Beijing Anzhen Nanchong Hospital, Capital Medical University & Nanchong Central Hospital, Nanchong, Sichuan, China; 4 Department of Orthopedics, Neijiang Hospital of Traditional Chinese Medicine, Neijiang, Sichuan, China

**Keywords:** digital pathology, machine learning, neoadjuvant therapy, pathologic complete response, spatial architecture, triple-negative breast cancer, tumor immune microenvironment, tumor-infiltrating lymphocytes

## Abstract

**Background:**

Stromal tumor-infiltrating lymphocytes (TILs) are established biomarkers in triple-negative breast cancer (TNBC), but conventional density-based assessment does not capture their spatial relationship to tumor nests. We evaluated whether an H&E-derived spatial architecture index (SAI) provides predictive information beyond stromal TIL density for pathologic complete response (pCR) after neoadjuvant therapy.

**Methods:**

This single-center retrospective cohort included 236 patients with TNBC who had evaluable pretreatment core biopsy slides and definitive surgical response assessment. The primary SAI was the equally weighted mean of standardized close interaction ratio and lymphocyte cluster index, together with reverse-coded standardized lymphocyte–tumor distance and edge enrichment. The primary multivariable analysis included 231 patients with complete clinicopathologic data. Robustness was evaluated using bootstrap optimism correction, alternative SAI constructions, treatment-regimen and propensity-score analyses, repeated nested cross-validation, and a 60-case reproducibility assessment. During internal validation, preprocessing and SAI construction were repeated within each training fold.

**Results:**

Of 236 patients, 122 (51.7%) achieved pCR. In the main multivariable model, the SAI remained associated with pCR (OR 2.79 per 1 SD, 95% CI 1.79–4.35; *P* < 0.001), whereas stromal TIL density was not independently significant (OR 0.88 per 10% increase, 95% CI 0.74–1.04; *P* = 0.123). Bootstrap optimism-corrected AUROCs were 0.698 for the clinical model, 0.705 after the addition of stromal TIL density, 0.764 after the addition of the SAI, and 0.765 after the addition of both. Across six internally validated algorithms using the full feature set, mean AUROCs ranged from 0.730 to 0.752, with only modest between-algorithm differences. The SAI showed excellent interobserver reproducibility (ICC 0.93, 95% CI 0.89–0.95). Its association with pCR persisted in the chemotherapy-only subgroup (OR 2.48, 95% CI 1.52–4.05; *P* < 0.001), whereas the interaction with pembrolizumab-containing treatment was not significant (*P* = 0.222).

**Conclusion:**

In this exploratory single-center retrospective cohort, the H&E-derived SAI provided internally validated predictive information beyond stromal TIL density for pCR. External multicenter validation, assessment of intersite technical reproducibility, and prospective calibration are required before clinical implementation.

## 1 Introduction

Triple-negative breast cancer (TNBC) is characterized by substantial biological heterogeneity, an aggressive clinical course, and limited broadly applicable targeted treatment options (1, 2). Neoadjuvant systemic therapy has become integral to the management of early-stage TNBC because it can facilitate surgical treatment and provide an *in vivo* measure of treatment sensitivity (3–5). Pathologic complete response (pCR) is particularly relevant in this setting, as pooled analyses have shown that its association with long-term outcomes is strongest in biologically aggressive subtypes, including TNBC (6). The incorporation of neoadjuvant chemoimmunotherapy has further increased the clinical importance of reliable pretreatment response stratification while adding complexity to treatment selection.

Among candidate biomarkers, tumor-infiltrating lymphocytes (TILs) are among the most reproducible and clinically informative in TNBC. Higher baseline stromal TIL levels have consistently been associated with a greater probability of pCR after neoadjuvant therapy and with more favorable survival outcomes (7–12). This evidence has supported international standardization of stromal TIL assessment on routine hematoxylin–eosin (H&E) sections (13–15). However, the simplicity of a single density estimate is also a limitation: stromal TIL percentage does not fully characterize the distribution of lymphocytes relative to tumor nests, stromal compartments, or the invasive interface (15, 16). Consequently, tumors with similar stromal TIL percentages may exhibit markedly different immune spatial organization.

This distinction may be biologically relevant. H&E morphology cannot identify specific immune-cell subsets or directly quantify immune function, but lymphocyte proximity, clustering, and localization at the tumor–stroma interface may reflect aspects of tumor–immune organization that are not captured by density alone (16, 17). A lymphocyte-rich but spatially excluded tumor is morphologically distinct from a tumor in which lymphocytes closely approach tumor nests. Spatial pathology studies have suggested that immune proximity, clustering, and tumor–stroma organization may provide prognostic or predictive information beyond cell abundance; however, much of the available evidence has focused on survival endpoints, mixed breast cancer populations, or technically complex platforms with limited routine applicability (17–21).

Digital pathology increasingly permits spatial immune features to be extracted directly from routine H&E slides. Pretreatment core biopsies are already obtained in standard clinical care, and H&E-based methods are more scalable than approaches that require multiplex staining, specialized imaging platforms, or high-cost spatial-omics workflows (19, 22–25). Computational studies in TNBC have used whole-slide imaging, stromal texture, and machine learning to predict neoadjuvant response, indicating that pretreatment histomorphology contains clinically relevant response information. Nevertheless, many existing models operate primarily as black-box predictors or emphasize global stromal and tissue-level features, creating a gap between predictive performance and biological interpretability (20–25). This gap is important for translational pathology because clinically useful biomarkers must be both technically feasible and interpretable to pathologists and oncologists.

An unresolved question is whether an H&E-derived spatial immune morphology metric can improve response prediction in TNBC beyond stromal TIL density. This question is particularly relevant in pretreatment biopsy specimens, in which tissue is limited and treatment has not yet begun. Although pembrolizumab-containing regimens have improved outcomes in early-stage TNBC, pathology-based tools for pretreatment response stratification remain limited. An interpretable spatial metric derived from routine H&E sections could therefore complement conventional clinicopathologic assessment without requiring an additional assay platform.

Accordingly, we integrated conventional stromal TIL assessment, digital spatial descriptors, representative spatial phenotypes, and internally validated prediction modeling in a retrospective neoadjuvant TNBC cohort. We hypothesized that the spatial organization of lymphocytes relative to tumor nests would provide predictive information beyond TIL density alone and could be quantified from routine pretreatment biopsy slides. We evaluated whether a composite SAI and related spatial immune descriptors were associated with pCR, whether the SAI provided incremental predictive information beyond clinicopathologic variables and stromal TIL density, and whether this contribution remained evident across multiple machine learning algorithms.

## 2 Methods

### 2.1 Study design and patient selection

This single-center retrospective cohort study included patients with TNBC who received neoadjuvant systemic therapy at a tertiary hospital between January 2019 and December 2025. Reporting followed the Strengthening the Reporting of Observational Studies in Epidemiology (STROBE) statement and the Transparent Reporting of a Multivariable Prediction Model for Individual Prognosis or Diagnosis (TRIPOD) statement (26, 27).

The Ethics Committee of The Second Hospital & Clinical Medical School, Lanzhou University approved the study (approval No. Lu2501223). The requirement for written informed consent was waived because of the retrospective design and the use of de-identified clinicopathologic data. The study was conducted in accordance with the Declaration of Helsinki.

Patients were eligible if they had (1) histologically confirmed TNBC in the pretreatment core biopsy specimen, (2) completed neoadjuvant systemic therapy followed by surgery, (3) an available pretreatment H&E slide suitable for digital pathology analysis, and (4) a definitive postoperative response assessment. Of 412 screened patients, 176 were excluded because pretreatment biopsy tissue was unavailable (*n* = 47), severe slide-quality limitations precluded digital analysis (*n* = 39), key clinicopathologic data were missing (*n* = 32), the neoadjuvant regimen was non-standard or treatment records were incomplete (*n* = 34), or definitive postoperative pCR assessment was unavailable at database lock (*n* = 24). The final analytic cohort comprised 236 patients. Available pretreatment characteristics were compared between included and excluded patients to evaluate potential selection bias (**Supplementary Tables 1**, **S2**; **Supplementary Figure 1**).

TNBC was defined from the institutional diagnostic pathology record by the absence of estrogen receptor and progesterone receptor expression and the absence of HER2 overexpression or amplification. The primary outcome was pCR, defined as no residual invasive carcinoma in the breast or sampled axillary lymph nodes, with residual *in situ* disease permitted (ypT0/is ypN0) (6).

### 2.2 Clinicopathologic variables and treatment-related data

Clinicopathologic data were retrieved from the electronic medical record and pathology database. Variables included age, body mass index, menopausal status, clinical T stage, clinical nodal status, histologic type, histologic grade, Ki-67, programmed death-ligand 1 (PD-L1) combined positive score (CPS), tertiary lymphoid structure (TLS) status, diagnosis year, neoadjuvant regimen, dose-reduction status, and digital slide-quality category. PD-L1 CPS was abstracted from institutional diagnostic pathology reports. TLS positivity was defined morphologically on H&E sections as organized lymphoid aggregates with features compatible with follicle formation in tumor-associated or peritumoral stroma.

Variables were harmonized before regression and prediction modeling. Clinical T stage was dichotomized as cT1–2 versus cT3–4, clinical nodal status as cN0 versus cN+, and histologic grade as grade 2 versus grade 3. Ki-67 and pathologist-read stromal TIL density were modeled per 10% increase. Neoadjuvant treatment was classified into three documented regimen groups: anthracycline/taxane, anthracycline/taxane plus carboplatin, and pembrolizumab plus carboplatin/taxane. Dose reduction was abstracted from treatment records. For subgroup analyses, age was categorized as <50 versus ≥50 years and PD-L1 CPS as <10 versus ≥10.

### 2.3 Histopathologic review, slide selection, and digital pathology workflow

Archived 4-μm pretreatment core biopsy H&E sections were retrieved for all eligible patients. When multiple biopsy levels were available, one representative slide containing invasive carcinoma and adequate tumor-associated stroma was selected. Slides were scanned with a Leica Aperio AT2 whole-slide scanner at ×40 magnification (native resolution, 0.25 μm/pixel) and analyzed at a ×20 equivalent resolution of 0.50 μm/pixel in SVS format. QuPath v0.5.1 and OpenSlide v4.0.0 were used for image review and processing, and R v4.3.2 was used for spatial and statistical analyses. Macenko-based H&E stain normalization was applied before batch feature extraction (**Supplementary Table 3**).

Tissue detection used an optical-density threshold of 0.15 and required at least 1.0 mm² of analyzable tissue and 0.5 mm² of tumor-associated stroma. Necrosis, crush artifact, pen marks, tissue folds, out-of-focus regions, benign breast tissue, ductal carcinoma *in situ*, and other non-invasive structures were excluded. Invasive tumor nests and associated stroma were manually annotated or verified by two breast pathologists who were blinded to pCR status and treatment regimen.

Pathologist-read stromal TILs were scored according to International TILs Working Group recommendations and related practical guidance (13–15, 28). Assessment was restricted to stromal areas within the borders of invasive carcinoma and excluded necrosis, artifacts, benign tissue, and *in situ* disease. Stromal TIL density was recorded as the percentage of intratumoral stromal area occupied by mononuclear inflammatory cells. Discordant or technically uncertain cases underwent joint review.

Nuclei were segmented with the StarDist 2D model using a probability threshold of 0.50 and a non-maximum suppression overlap threshold of 0.40. Lymphocytes were identified with a locked random forest classifier trained on 2,800 pathologist-labeled nuclei from 30 representative slides. In a held-out validation subset, classifier accuracy was 0.89, precision was 0.87, recall was 0.85, and the F1 score was 0.86. Classifier settings were locked before application to the analytic cohort.

Image quality was classified as pass, minor artifact, or segmentation review. Slides were excluded if severe blur, folds, crush artifact, or necrosis affected more than 30% of the analyzable tumor–stroma interface or if tumor/stroma annotation failed. Slides with limited artifacts but preserved interpretability were retained after manual review. Among the 236 analyzed slides, 194 passed quality control, 29 had minor artifacts, and 13 required segmentation review (**Supplementary Table 3**).

### 2.4 Spatial feature extraction and phenotype assignment

Following digital review, tumor nests, tumor-associated stroma, and the 0–50-μm tumor–stroma interface band were analyzed. The primary exposure was the SAI, a continuous composite designed to summarize lymphocyte proximity, local clustering, and edge restriction relative to tumor nests. Pathologist-read stromal TIL density and digital TIL area were retained as density descriptors but were not included in the primary SAI (**Supplementary Table 4**).

The primary SAI was calculated as SAIraw = [*z*(close interaction ratio) + *z*(lymphocyte cluster index) − *z*(mean lymphocyte − tumor distance) − *z*(edge enrichment score)]/4. Distance and edge enrichment were reverse-coded because longer distance and greater edge restriction indicate less tumor-proximal organization. For the primary regression analysis, the seven missing close-interaction values were replaced with the cohort median; the other three components were complete. The equally weighted composite was then restandardized to a mean of 0 and an SD of 1. Cohort-level standardization parameters are reported in **Supplementary Table 4**. For internal validation, all imputation, standardization, and SAI-construction parameters were estimated within each training fold and applied without modification to the corresponding held-out fold.

Digital TIL area was defined as the percentage of stromal area occupied by detected lymphocytes. Mean lymphocyte–tumor distance was calculated from each lymphocyte centroid to the nearest tumor boundary. The close interaction ratio was the proportion of lymphocytes located within 20 μm of the nearest tumor boundary. The cluster index summarized local lymphocyte aggregation within a 50-μm neighborhood, whereas edge enrichment quantified preferential localization within the 0–50-μm tumor–stroma interface band relative to the remaining stromal compartment. Component distributions and extreme values were verified against the corresponding whole-slide images.

For descriptive visualization, each case was assigned to one of four representative spatial phenotypes: immune-excluded, margin-restricted, inflamed-dispersed, or inflamed-proximal. Inflammation was characterized by stromal TIL density and digital TIL area, proximity by mean lymphocyte–tumor distance and close interaction ratio, and interface restriction by edge enrichment. Classification was based on the dominant pattern across the annotated invasive tumor and associated stroma. When multiple patterns coexisted, the phenotype occupying the largest analyzable tumor–stroma area was assigned after review. All 236 cases were classifiable; operational definitions and quantitative summaries are provided in **Supplementary Table 5**.

### 2.5 Reproducibility assessment

A reproducibility subset of 60 cases was selected with 1:1 stratification by pCR status. Two breast pathologists independently reassessed stromal TIL density, TLS status, spatial phenotype, image-quality category, and tumor–stroma annotations while blinded to pCR status, treatment regimen, and each other’s assessments. Spatial metrics were recalculated from the independently reviewed annotations. Continuous measurements were evaluated with a two-way random-effects, absolute-agreement, single-measure intraclass correlation coefficient [ICC (2,1)], and categorical assessments with Cohen’s *κ*. Detailed agreement estimates are reported in **Supplementary Table 6**.

### 2.6 Statistical analysis

All analyses were performed in R v4.3.2. Continuous variables were summarized as mean ± SD or median (interquartile range), as appropriate, and categorical variables as *n* (%). Continuous variables were compared with the Wilcoxon rank-sum test, and categorical variables with the chi-square test or Fisher’s exact test. No outcome data were missing. Missingness was limited to PD-L1 CPS (*n* = 14), close interaction ratio (*n* = 7), body mass index (*n* = 6), digital TIL area (*n* = 6), and Ki-67 (*n* = 5). The primary regression analysis used complete clinicopathologic covariates (*n* = 231); missing values for one spatial component were median-imputed only for construction of the primary SAI, with a complete-case SAI analysis conducted as a sensitivity analysis. Missing-data counts and analysis denominators are summarized in **Supplementary Table 7**.

The primary inferential analysis used multivariable binary logistic regression with pCR as the dependent variable. The prespecified model included age, clinical T stage, clinical nodal status, histologic grade, Ki-67, neoadjuvant regimen, stromal TIL density, and the SAI. Odds ratios (ORs) with 95% confidence intervals (CIs) were reported. Robustness to SAI construction was evaluated using complete-case, leave-one-component-out, rank-based, principal-component-derived, and exploratory six-descriptor indices (**Supplementary Table 8**). A separate sensitivity model was additionally adjusted for TLS.

Potential treatment-related confounding was evaluated using separate chemotherapy-only and pembrolizumab-containing subgroup models, an SAI × pembrolizumab-containing treatment interaction, additional adjustment for diagnosis year and treatment period (2019–2022 versus 2023–2025), and propensity-score analyses with logit adjustment, inverse probability of treatment weighting, and overlap weighting. The propensity model included age, clinical T stage, clinical nodal status, grade, Ki-67, stromal TIL density, SAI, PD-L1 CPS, TLS, and diagnosis year (**Supplementary Table 9**).

The association between SAI and stromal TIL density was characterized by complete-case Spearman correlation. Collinearity was assessed with adjusted variance inflation factors, and the stromal TIL coefficient was compared before and after inclusion of the SAI (**Supplementary Table 10**).

Incremental predictive performance was evaluated with four sequential logistic models fitted to the same analysis sample: a clinical model, a clinical model plus stromal TIL density, a clinical model plus SAI, and a clinical model plus both stromal TIL density and SAI. Apparent area under the receiver operating characteristic curve (AUROC), area under the precision–recall curve (AUPRC), Brier score, calibration intercept, and calibration slope were calculated. Optimism was estimated from 500 bootstrap resamples, and optimism-corrected performance was reported for all four models (**Supplementary Table 11**). All tests were two-sided, and *P* < 0.05 was considered statistically significant.

### 2.7 Machine learning modeling and internal validation

To complement the regression analyses, we developed and internally validated six machine learning models for pCR prediction: logistic regression, ridge regression, Elastic Net, linear support vector machine (SVM), random forest, and extreme gradient boosting (XGBoost). Three prespecified feature sets were evaluated. FS1 contained clinicopathologic variables only (age, clinical T stage, clinical nodal status, histologic grade, Ki-67, and neoadjuvant regimen); FS2 added pathologist-read stromal TIL density; and FS3 further added the SAI.

All preprocessing was confined to the resampling framework. Numeric imputation and normalization, categorical imputation and dummy encoding, zero-variance filtering, and construction and standardization of the four-component SAI were estimated within each training partition and then applied unchanged to the held-out partition. Internal validation used the outer 5-fold cross-validation repeated 10 times, with inner 5-fold cross-validation for hyperparameter tuning. Logistic regression was fitted without tuning; ridge regression, Elastic Net, linear SVM, random forest, and XGBoost were tuned using AUROC as the optimization metric.

Performance was summarized across 50 outer resamples as mean ± SD and 95% empirical intervals for AUROC, together with AUPRC and Brier score. Calibration intercept and slope were calculated from averaged out-of-fold predicted probabilities. Decision curve analysis quantified net benefit across threshold probabilities from 0.05 to 0.50 (29). Performance was summarized for the full feature set, across all prespecified feature sets, and at selected decision-curve thresholds.

## 3 Results

### 3.1 Patient cohort and baseline characteristics

**Figure 1:**
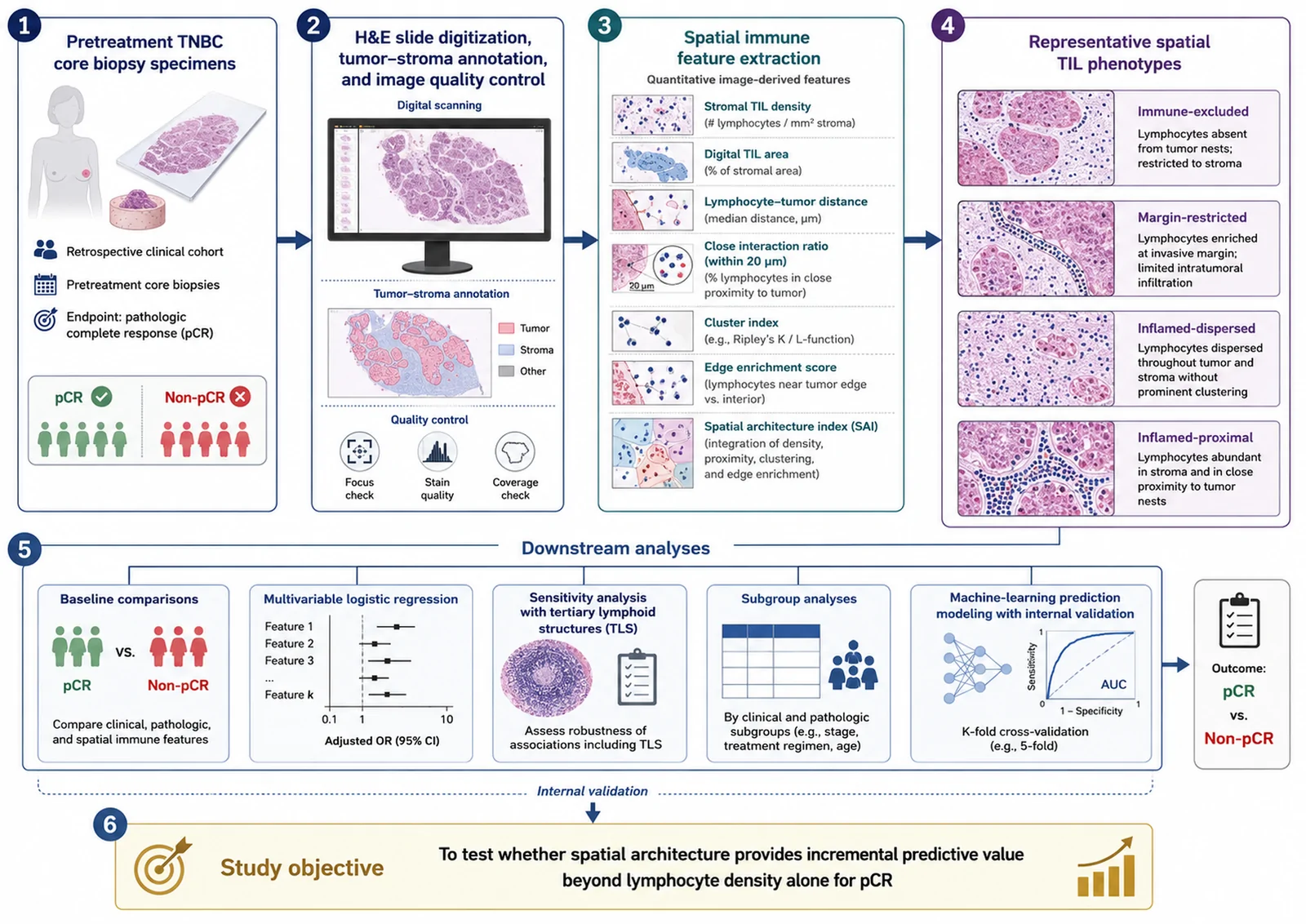
Analytic workflow for spatial immune characterization and predictive modeling in pretreatment TNBC biopsy specimens. Pretreatment core biopsy H&E slides were scanned, annotated, and subjected to image-quality control. Measured descriptors included pathologist-read stromal TIL density, digital TIL area, lymphocyte–tumor distance, close interaction ratio within 20 μm, cluster index, and edge enrichment. The primary SAI was an equally weighted composite of close interaction, clustering, reverse-coded distance, and reverse-coded edge enrichment; density measures were not included in the primary SAI. Downstream analyses comprised multivariable regression, sensitivity and treatment-confounding analyses, and repeated nested cross-validation for pCR prediction.

The study workflow is shown in **Figure 1** and **Supplementary Figure 1**. Of 412 screened patients, 236 (57.3%) were included in the analytic cohort and 176 were excluded: 47 lacked pretreatment biopsy tissue, 39 had severe slide-quality limitations, 32 had missing key clinicopathologic data, 34 had a non-standard neoadjuvant regimen or incomplete treatment records, and 24 lacked definitive postoperative pCR assessment. The final cohort included 122 patients with pCR (51.7%) and 114 without pCR. Available pretreatment characteristics were similar between included and excluded patients, including age (48.6 ± 9.9 vs. 49.1 ± 10.1 years; *P* = 0.607), Ki-67 (65.3% ± 15.8% vs. 64.8% ± 15.1%; *P* = 0.503), and neoadjuvant regimen among evaluable records (*P* = 0.537) (**Supplementary Tables 1**, **S2**).

Baseline characteristics are summarized in **Table 1**. Age, body mass index, menopausal status, clinical nodal status, histologic type, PD-L1 CPS, dose reduction, and image-quality category did not differ significantly by response status. Compared with the non-pCR group, the pCR group had fewer cT3 tumors (16% vs. 38%; overall T-stage distribution *P* < 0.001), a higher proportion of grade 3 tumors (89% vs. 74%; *P* = 0.003), higher mean Ki-67 (67.8% vs. 62.7%; *P* = 0.010), more frequent TLS positivity (46% vs. 33%; *P* = 0.049), and greater use of pembrolizumab plus carboplatin/taxane (36% vs. 22%; overall regimen distribution *P* = 0.041). The pCR group also had higher stromal TIL density (39.0% ± 25.0% vs. 31.0% ± 21.4%; *P* = 0.014), a shorter mean lymphocyte–tumor distance (38.7 ± 8.9 vs. 44.4 ± 8.7 μm; *P* < 0.001), lower edge enrichment (56.50 ± 21.15 vs. 71.12 ± 19.94; *P* < 0.001), and a higher standardized SAI (0.34 ± 0.94 vs. −0.36 ± 0.93; *P* < 0.001) (**Supplementary Figure 2**). These findings indicate that pCR was associated not only with greater lymphocyte abundance but also with a more tumor-proximal and less edge-restricted spatial pattern.

**Table 1:** Baseline characteristics of the study cohort according to pathologic complete response status.

Variable	Overall	Non-pCR	pCR	P-value
Age, years	48.6 ± 9.9	47.7 ± 10.3	49.5 ± 9.4	0.085
Body mass index, kg/m²	24.4 ± 3.6	24.3 ± 3.6	24.6 ± 3.6	0.600
Menopausal status				0.500
Pre	131 (56%)	66 (58%)	65 (53%)	
Post	105 (44%)	48 (42%)	57 (47%)	
Clinical T stage				<0.001
cT1	28 (12%)	12 (11%)	16 (13%)	
cT2	140 (59%)	55 (48%)	85 (70%)	
cT3	63 (27%)	43 (38%)	20 (16%)	
cT4	5 (2.1%)	4 (3.5%)	1 (0.8%)	
Clinical nodal status				0.110
cN0	115 (49%)	48 (42%)	67 (55%)	
cN1	84 (36%)	44 (39%)	40 (33%)	
cN2–3	37 (16%)	22 (19%)	15 (12%)	
Histologic type				0.700
IDC	202 (86%)	98 (86%)	104 (85%)	
Medullary-like	13 (5.5%)	5 (4.4%)	8 (6.6%)	
Metaplastic	4 (1.7%)	3 (2.6%)	1 (0.8%)	
Other	17 (7.2%)	8 (7.0%)	9 (7.4%)	
Histologic grade				0.003
2	44 (19%)	30 (26%)	14 (11%)	
3	192 (81%)	84 (74%)	108 (89%)	
Ki-67, %	65.3 ± 15.8	62.7 ± 16.8	67.8 ± 14.5	0.010
PD-L1 CPS	10.7 ± 8.5	9.6 ± 8.0	11.6 ± 8.9	0.100
TLS present	94 (40%)	38 (33%)	56 (46%)	0.049
Neoadjuvant regimen				0.041
Anthracycline/taxane	104 (44%)	58 (51%)	46 (38%)	
Anthracycline/taxane + carboplatin	63 (27%)	31 (27%)	32 (26%)	
Pembrolizumab + carboplatin/taxane	69 (29%)	25 (22%)	44 (36%)	
Dose reduction	42 (18%)	17 (15%)	25 (20%)	0.300
Image quality control				0.200
Pass	194 (82%)	89 (78%)	105 (86%)	
Minor artifact	29 (12%)	18 (16%)	11 (9.0%)	
Segmentation review	13 (5.5%)	7 (6.1%)	6 (4.9%)	
Pathologist-read stromal TILs, %	35.1 ± 23.6	31.0 ± 21.4	39.0 ± 25.0	0.014
Mean lymphocyte–tumor distance, μm	41.5 ± 9.2	44.4 ± 8.7	38.7 ± 8.9	<0.001
Edge enrichment score	63.56 ± 21.80	71.12 ± 19.94	56.50 ± 21.15	<0.001
Spatial architecture index, standardized	0.00 ± 1.00	−0.36 ± 0.93	0.34 ± 0.94	<0.001
Spatial phenotype				<0.001
Immune-excluded	62 (26%)	40 (35%)	22 (18%)	
Margin-restricted	67 (28%)	37 (32%)	30 (25%)	
Inflamed-dispersed	65 (28%)	25 (22%)	40 (33%)	
Inflamed-proximal	42 (18%)	12 (11%)	30 (25%)	

Values are presented as mean ± SD or *n* (%), as appropriate. *P*-values were calculated using the Wilcoxon rank-sum test for continuous variables and the chi-square test or Fisher’s exact test for categorical variables. PD-L1 values are based on available cases. The SAI was standardized internally to a mean of 0 and an SD of 1; raw component distributions are provided in **Supplementary Tables 4**, **S5**.

CPS, combined positive score; PD-L1, programmed death-ligand 1; pCR, pathologic complete response; TILs, tumor-infiltrating lymphocytes; TLS, tertiary lymphoid structures.

### 3.2 Spatial phenotype classification and reproducibility

**Figure 2:**
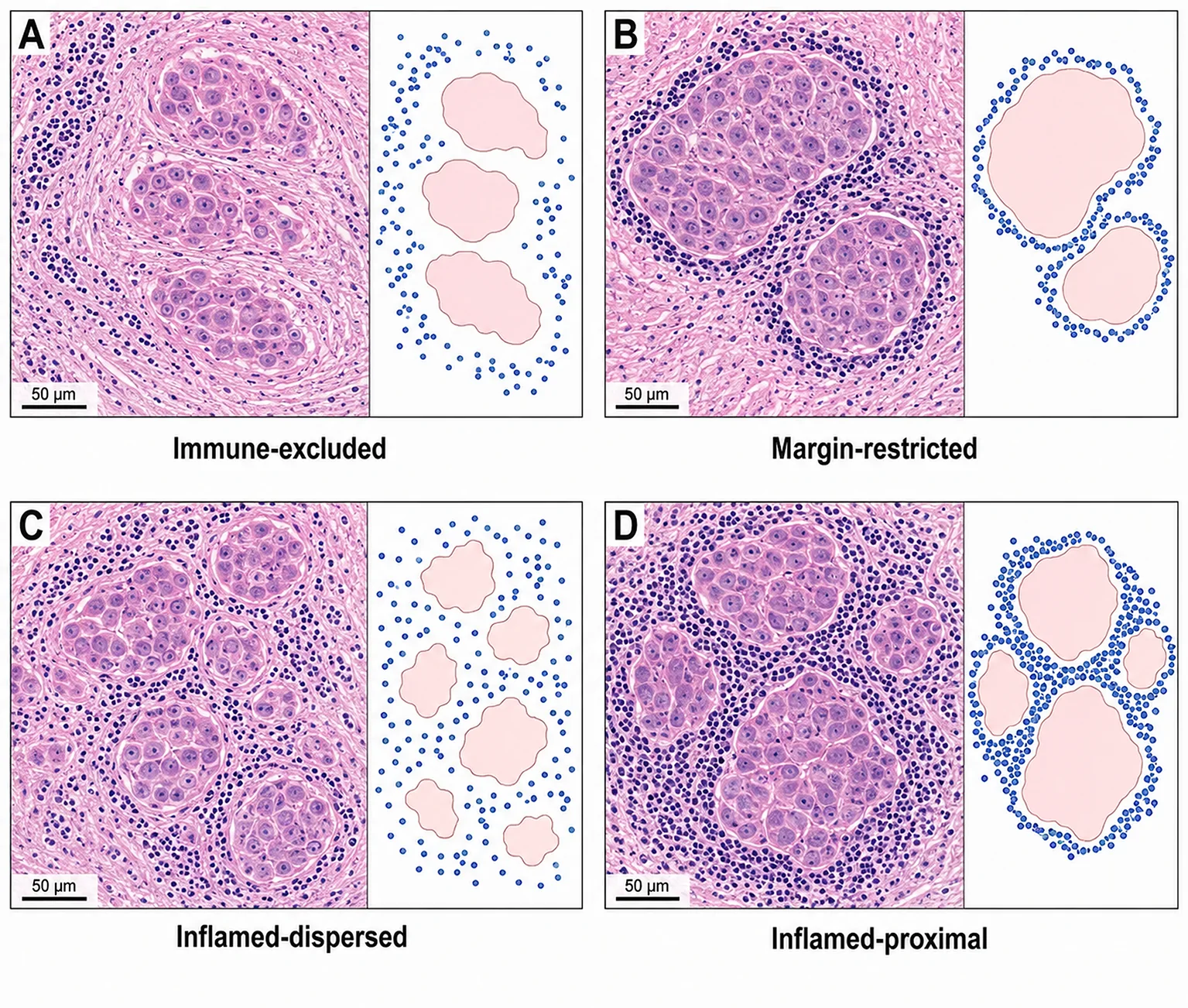
Representative dominant spatial TIL phenotypes in pretreatment TNBC biopsy specimens. H&E images and schematic illustrations show **(A)** immune-excluded, **(B)** margin-restricted, **(C)** inflamed-dispersed, and **(D)** inflamed-proximal patterns. Phenotypes were assigned according to the dominant pattern across annotated invasive tumor and tumor-associated stroma using descriptors of inflammation, proximity, clustering, and edge restriction. When mixed patterns were present, the phenotype occupying the largest analyzable area was assigned. Operational definitions and quantitative summaries are provided in **Supplementary Table 5**. Scale bars, 50 μm.

All 236 cases were assigned to a dominant spatial phenotype (**Figure 2**). The pCR rate increased descriptively across the immune-excluded, margin-restricted, inflamed-dispersed, and inflamed-proximal phenotypes: 35.5% (22/62), 44.8% (30/67), 61.5% (40/65), and 71.4% (30/42), respectively. Across the same phenotypes, median SAI increased from −0.87 to −0.46, 0.44, and 1.29; median lymphocyte–tumor distance decreased from 48.4 to 44.5, 36.9, and 29.1 μm; and median edge enrichment decreased from 84.8 to 76.3, 51.1, and 33.5. These descriptive gradients were consistent with the operational phenotype definitions (**Supplementary Table 5**).

Interobserver reproducibility was good to excellent (**Supplementary Table 6**). The SAI had an ICC of 0.93 (95% CI 0.89–0.95), and ICCs for its component spatial measures ranged from 0.90 to 0.97. Agreement for the four-category spatial phenotype was 85.0% (*κ* = 0.79, 95% CI 0.66–0.91). Cohen’s *κ* was 0.83 (95% CI 0.66–0.96) for TLS status and 0.75 (95% CI 0.46–0.94) for image-quality category.

### 3.3 Spatial architecture was independently associated with pCR

In the primary multivariable analysis of 231 complete cases (**Table 2**), the SAI remained associated with pCR after adjustment for clinicopathologic variables, treatment regimen, and stromal TIL density (OR 2.79 per 1 SD, 95% CI 1.79–4.35; *P* < 0.001). Clinical T stage cT3–4 was inversely associated with pCR (OR 0.46, 95% CI 0.22–0.93; *P* = 0.030), whereas grade 3 histology (OR 2.58, 95% CI 1.06–6.29; *P* = 0.037) and pembrolizumab plus carboplatin/taxane (OR 3.12, 95% CI 1.47–6.62; *P* = 0.003) were positively associated. Age, clinical nodal status, Ki-67, and anthracycline/taxane plus carboplatin were not independently associated with pCR. Stromal TIL density was also not independently significant after inclusion of the SAI (OR 0.88 per 10% increase, 95% CI 0.74–1.04; *P* = 0.123). In a model additionally adjusted for TLS, the SAI estimate was essentially unchanged (OR 2.81, 95% CI 1.78–4.45; *P* < 0.001), whereas TLS was not independently associated with pCR (OR 0.96, 95% CI 0.49–1.87; *P* = 0.906) (**Supplementary Table 12**).

**Table 2:** Multivariable logistic regression analysis for pathologic complete response.

Variable	Odds ratio (95% CI)	P-value
Age (per year)	1.02 (0.99 to 1.05)	0.297
Clinical T stage, cT3–4 vs. cT1–2	0.46 (0.22 to 0.93)	0.030
Clinical nodal status, cN+ vs. cN0	0.72 (0.39 to 1.35)	0.307
Histologic grade, 3 vs. 2	2.58 (1.06 to 6.29)	0.037
Ki-67 (per 10%)	1.18 (0.96 to 1.46)	0.122
Anthracycline/taxane + carboplatin vs. anthracycline/taxane	1.44 (0.69 to 2.98)	0.330
Pembrolizumab + carboplatin/taxane vs. anthracycline/taxane	3.12 (1.47 to 6.62)	0.003
Pathologist-read stromal TILs (per 10%)	0.88 (0.74 to 1.04)	0.123
Spatial architecture index (per 1 SD)	2.79 (1.79 to 4.35)	<0.001

The analytic sample included 231 patients with complete clinicopathologic covariates. ORs were estimated using multivariable binary logistic regression. Ki-67 and stromal TIL density were modeled per 10% increase, and the SAI was modeled per 1 SD. Reference categories were cT1–2, cN0, histologic grade 2, and the anthracycline/taxane regimen.

CI, confidence interval; cN, clinical nodal stage; cT, clinical tumor stage; OR, odds ratio; pCR, pathologic complete response; SD, standard deviation; TILs, tumor-infiltrating lymphocytes.

The SAI was moderately correlated with stromal TIL density (Spearman *ρ* = 0.589; *P* < 0.001), while the maximum adjusted variance inflation factor in the primary model was 1.32. The stromal TIL OR changed from 1.12 (95% CI 0.99–1.27; *P* = 0.077) before SAI inclusion to 0.88 (95% CI 0.74–1.04; *P* = 0.123) after SAI inclusion. Together with the broader correlation matrix, these findings support overlapping predictive information between lymphocyte abundance and spatial organization without evidence of problematic multicollinearity (**Supplementary Tables 10**, **S13**).

### 3.4 Spatial architecture added predictive value beyond density alone

Sequential model performance is shown in **Table 3** and **Supplementary Table 11**. Apparent AUROC increased from 0.730 for the clinical model to 0.740 after the addition of stromal TIL density, 0.794 after the addition of the SAI, and 0.797 after the addition of both. After bootstrap optimism correction, the corresponding AUROCs were 0.698, 0.705, 0.764, and 0.765. Relative to the clinical model, the absolute corrected AUROC gain was 0.007 with stromal TIL density alone, 0.066 with the SAI alone, and 0.067 with both variables.

**Table 3:** Apparent and bootstrap optimism-corrected performance of sequential logistic models for predicting pathologic complete response.

Model	Apparent AUROC	Corrected AUROC	Corrected AUPRC	Corrected Brier	Calibration intercept	Calibration slope
Clinical model	0.730	0.698	0.689	0.226	−0.001	0.840
Clinical + TIL density	0.740	0.705	0.686	0.224	0.001	0.825
Clinical + SAI	0.794	0.764	0.764	0.203	−0.000	0.855
Clinical + TIL density + SAI	0.797	0.765	0.771	0.203	0.001	0.839

Performance was estimated in 231 patients. Optimism-corrected estimates were obtained from 500 bootstrap resamples. Lower Brier scores indicate lower prediction error; calibration intercepts and slopes closer to 0 and 1, respectively, indicate better calibration.

AUROC, area under the receiver operating characteristic curve; AUPRC, area under the precision–recall curve; pCR, pathologic complete response; SAI, spatial architecture index; TILs, tumor-infiltrating lymphocytes.

The corrected AUPRC values were 0.689, 0.686, 0.764, and 0.771 across the same four models, while corrected Brier scores were 0.226, 0.224, 0.203, and 0.203, respectively. Calibration slopes were 0.840 for the clinical model, 0.825 after the addition of density, 0.855 after the addition of the SAI, and 0.839 after the addition of both. These descriptive comparisons indicate that most of the incremental predictive information was associated with spatial architecture rather than stromal TIL density alone; no formal between-model hypothesis test was performed.

### 3.5 Robustness and treatment-confounding analyses

The primary SAI result was stable across alternative constructions (**Supplementary Table 8**). In the complete-case four-component analysis (*n* = 224), the OR was 2.74 (95% CI 1.76–4.28; *P* < 0.001). Across leave-one-component-out, rank-based, and principal-component-derived indices, ORs ranged from 2.62 to 2.79 (all *P* < 0.001). An exploratory six-descriptor index that incorporated both spatial and density measures also remained associated with pCR (OR 1.99, 95% CI 1.42–2.79; *P* < 0.001).

The SAI remained associated with pCR in the chemotherapy-only subgroup (*n* = 165; OR 2.48, 95% CI 1.52–4.05; *P* < 0.001) and the pembrolizumab-containing subgroup (*n* = 66; OR 5.23, 95% CI 1.75–15.66; *P* = 0.003). However, the SAI × pembrolizumab-containing treatment interaction was not significant (interaction OR 1.75, 95% CI 0.71–4.32; *P* = 0.222), providing no evidence of treatment-effect modification. The association was similar after adjustment for diagnosis year (OR 2.82, 95% CI 1.81–4.41) or treatment period (OR 2.80, 95% CI 1.80–4.36) and in propensity-score logit-adjusted, inverse-probability-weighted, and overlap-weighted analyses (OR range 2.61–3.14; all *P* < 0.001). After overlap weighting, the largest absolute standardized mean difference was 0.064 (**Supplementary Table 9**).

### 3.6 Machine learning validation and model-based importance

**Figure 3:**
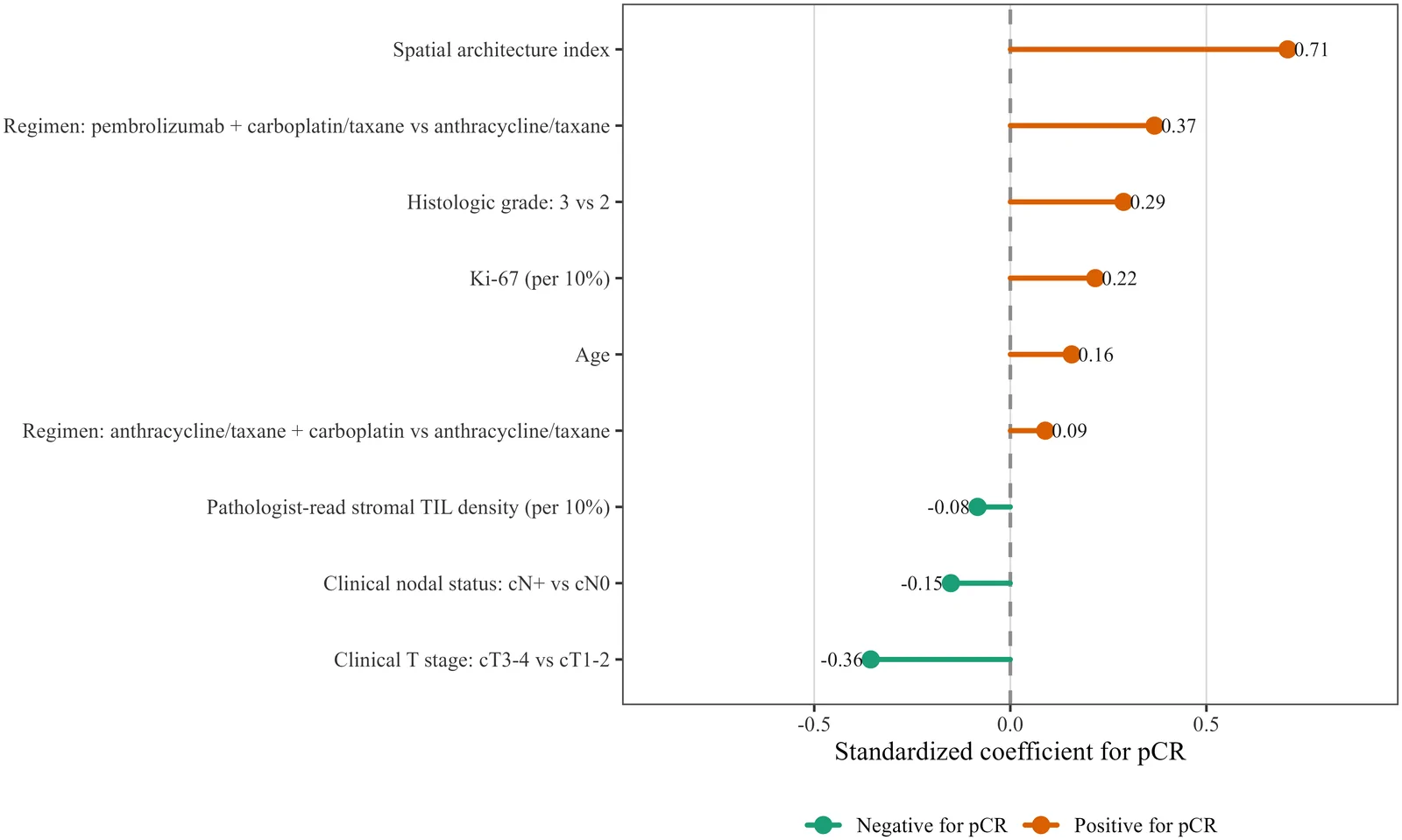
Model-based standardized coefficients from the final elastic net model. Standardized coefficients are shown for the full feature set. For visualization, coefficient signs were oriented so that positive values corresponded to a higher predicted probability of pCR and negative values to a higher predicted probability of non-pCR. Coefficient magnitude represents model contribution and should not be interpreted causally. The SAI had the largest coefficient magnitude.

Model-based standardized coefficients from the final Elastic Net model are shown in **Figure 3**. Coefficient signs were oriented so that positive values corresponded to a higher predicted probability of pCR. The SAI had the largest coefficient magnitude, followed by pembrolizumab-containing treatment and clinical T stage. These coefficients quantify contribution within the prediction model and should not be interpreted as causal effects.

Across the prespecified feature sets, mean AUROCs ranged from 0.675 to 0.689 for FS1 (clinical variables), from 0.676 to 0.702 for FS2 (clinical variables plus stromal TIL density), and from 0.730 to 0.752 for FS3 (clinical variables, stromal TIL density, and SAI) (**Supplementary Table 14**; **Supplementary Figure 4**). With the full feature set, logistic regression achieved a mean AUROC of 0.752 ± 0.066 (95% empirical interval 0.629–0.877) and a Brier score of 0.207 ± 0.030 (**Table 4**). Elastic Net produced a similar AUROC of 0.751 ± 0.065 and Brier score of 0.207 ± 0.024, with a calibration slope of 0.998. Ridge regression, linear SVM, and random forest showed comparable discrimination, whereas XGBoost was modestly lower (AUROC 0.730 ± 0.072). The substantial overlap in empirical intervals indicated limited evidence of meaningful algorithm-level differences.

**Table 4:** Performance of six machine learning models for predicting pathologic complete response using the full feature set.

Model	AUROC, mean ± SD	95% empirical interval	AUPRC, mean ± SD	Brier, mean ± SD	Calibration intercept	Calibration slope	Resamples
Logistic regression	0.752 ± 0.066	0.629 to 0.877	0.769 ± 0.075	0.207 ± 0.030	−0.009	0.770	50
Elastic Net	0.751 ± 0.065	0.631 to 0.875	0.766 ± 0.072	0.207 ± 0.024	−0.013	0.998	50
Ridge regression	0.751 ± 0.064	0.637 to 0.867	0.767 ± 0.071	0.214 ± 0.021	−0.007	1.364	50
Linear SVM	0.749 ± 0.063	0.630 to 0.869	0.767 ± 0.068	0.208 ± 0.020	0.009	1.093	50
Random forest	0.746 ± 0.065	0.625 to 0.876	0.767 ± 0.070	0.212 ± 0.016	−0.006	1.651	50
XGBoost	0.730 ± 0.072	0.599 to 0.854	0.760 ± 0.077	0.226 ± 0.023	−0.011	1.575	50

The full feature set comprised clinicopathologic variables, stromal TIL density, and the SAI. AUROC, AUPRC, and Brier score are reported across 50 outer resamples from repeated nested cross-validation. Calibration intercept and slope were calculated from averaged out-of-fold predictions. Empirical intervals represent the 2.5th and 97.5th percentiles of outer-resample AUROCs.

AUROC, area under the receiver operating characteristic curve; AUPRC, area under the precision–recall curve; pCR, pathologic complete response; SVM, support vector machine; TIL, tumor-infiltrating lymphocyte.

**Figure 4:**
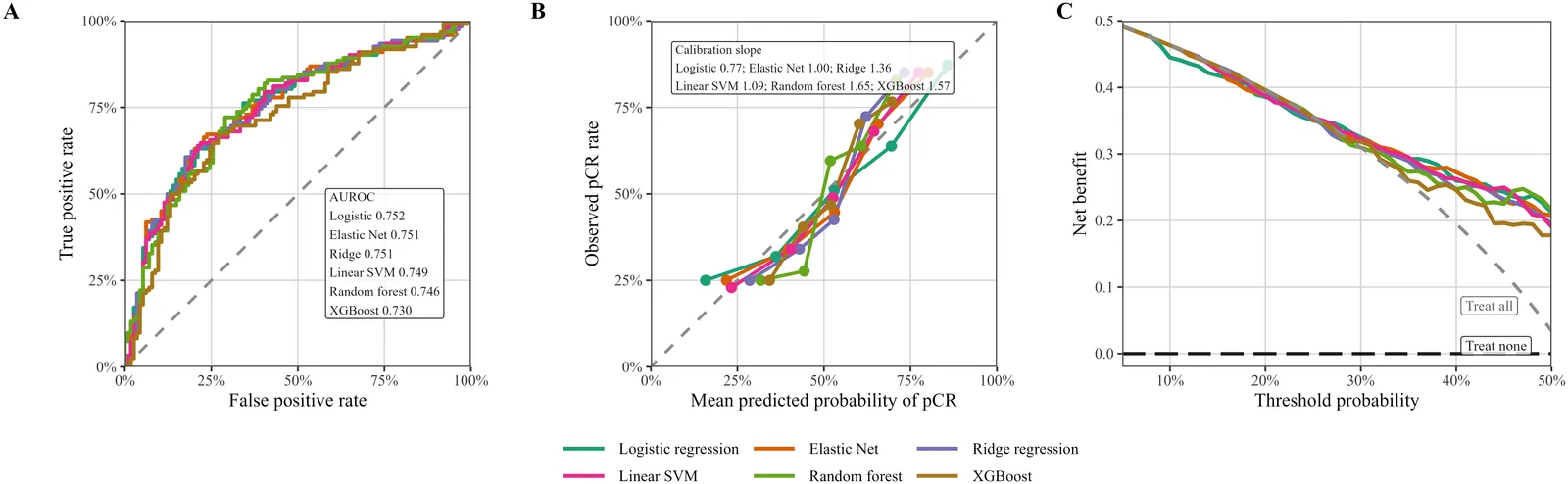
Internal validation of six prediction models using the full feature set. The full feature set included clinicopathologic variables, stromal TIL density, and the SAI. **(A)** Receiver operating characteristic curves derived from averaged out-of-fold predictions. **(B)** Calibration curves; model-specific calibration estimates are reported in **Table 4**. **(C)** Decision curve analysis across threshold probabilities from 0.05 to 0.50, with treat-all and treat-none reference strategies. The evaluated models were logistic regression, Elastic Net, ridge regression, linear SVM, random forest, and XGBoost.

Cross-validated ROC, calibration, and decision curves are presented in **Figure 4**. The decision curves were closely grouped across algorithms. At a threshold probability of 0.30, model net benefit ranged from 0.310 to 0.325; at a threshold of 0.40, it ranged from 0.246 to 0.273 (**Supplementary Table 15**). Thus, the full-feature models showed similar decision-analytic performance, without a clear clinical advantage for any single algorithm.

Fold-level distributions of AUROC, AUPRC, and Brier score are shown in **Supplementary Figure 3**. Across most algorithms, the principal performance gain occurred when spatial architecture was added to the clinical and density feature set, whereas the addition of stromal TIL density alone produced only a small change. This pattern was consistent across discrimination and prediction-error metrics, although all estimates remain internally validated and require external confirmation.

## 4 Discussion

Density and spatial organization are related, but they are not interchangeable descriptions of the immune infiltrate. A stromal TIL percentage tells us how much lymphocytic inflammation is present; it says much less about whether those cells remain at the margin or reach the tumor nests. This is the setting in which the SAI is most appropriately interpreted. It is not a substitute for conventional TIL scoring, and the present data do not support its use as a stand-alone clinical test. Rather, it is a way of asking whether routine H&E contains architectural information that is lost when immune infiltration is summarized by a single percentage.

That interpretation does not conflict with the established TNBC literature. Higher stromal TIL levels have repeatedly been associated with neoadjuvant response and favorable outcomes (7, 9, 10, 30). Indeed, the consensus scoring frameworks were designed to provide a reproducible measure of stromal immune infiltration, not a map of the geometric relation between lymphocytes and malignant epithelium (13, 14, 28). A density biomarker can therefore remain informative while leaving room for additional spatial information. Fisher et al. approached response prediction from another H&E-based direction and showed that pretreatment whole-slide tissue and texture features contained information about chemotherapy response (31). The present analysis addresses a narrower question: whether a small set of interpretable immune-topology features adds information beyond the conventional stromal percentage.

Evidence from more resolved spatial assays points in the same direction. In neoadjuvant TNBC, Wang et al. found that pretreatment cellular composition and multicellular organization were associated with immunotherapy response (32). Liang et al. reported that a higher proportion of CD8-positive T cells within 20 μm of tumor cells was associated with pCR and favorable survival outcomes across breast cancer subtypes (33). More recently, single-cell and spatial transcriptomic profiling identified TNBC cellular ecotypes whose composition and organization differed according to chemotherapy response (34). These studies measure cell identity and molecular state at a level that routine H&E cannot reproduce, so none should be taken as direct validation of the SAI. What they do provide is biological context for the same general observation: where cells are located, and which cells they are located next to, may matter in addition to how many are present. The question here is whether a useful fraction of that information can be recovered from ordinary biopsy morphology.

Biologically, the relevant distinction may be between an immune-rich tumor and an immune-accessible tumor. Work on host immunity and organized lymphoid structures supports the importance of immune-cell organization and interaction, not merely their presence (16, 35). The observation of Liang et al. that short-range CD8-positive T-cell proximity was associated with treatment response is particularly pertinent (33), because it provides a cell-specific example of the broader proximity concept used here. The analogy has limits: our H&E classifier does not identify CD8-positive cells, and a 20-μm morphologic proximity measure is not a functional assay. Likewise, the TNBC ecotypes described by spatial profiling include macrophage states, tumor-cell programs, interferon signaling, and HLA expression as well as lymphocytes (34). It is therefore plausible that the SAI reflects the morphologic footprint of a multicellular tissue state rather than one immune pathway. Reports that tumor–immune interface organization carries information beyond simple cell counts in computational pathology are consistent with that interpretation (36–38).

The apparent weakening of the stromal TIL coefficient after adding the SAI could otherwise be misread. It does not reverse the extensive evidence supporting stromal TILs in TNBC (9, 16). Density and spatial organization are correlated descriptors of the same microenvironment, so their coefficients in a joint model are conditional on one another. The low variance-inflation factors argue against severe multicollinearity, but shared information is expected. We therefore read the attenuation of the density coefficient as evidence that the SAI captures partly overlapping, and perhaps more topologically specific, information—not as evidence that greater lymphocyte infiltration is unfavorable.

A practical advantage of the SAI is that it starts from H&E, but that advantage should not be confused with readiness for use. H&E is already part of the diagnostic workflow and does not require additional tissue or instrumentation. Even so, a spatial metric will only be useful if it is reproducible outside the environment in which it was developed. The multicenter TIGER challenge showed that computational TIL assessment can be benchmarked across large breast cancer datasets while also underscoring the need for standardization before implementation (39). Other computational TIL studies similarly caution that algorithms and feature-extraction pipelines should not be assumed to be interchangeable (36, 38, 40, 41). Against that background, the lack of a clear advantage for a more complex algorithm in our data is reassuring only to a point. The more important next questions concern whether the spatial features themselves are stable across scanners and institutions, whether predictions remain calibrated, and whether the added information changes decisions beyond standardized TIL assessment.

The pembrolizumab and TLS analyses are better viewed as limits on interpretation than as separate positive findings. Spatially resolved TNBC studies show that immune organization can be related to checkpoint-blockade response (32), but this cohort was neither randomized by treatment nor designed to establish a pembrolizumab-specific biomarker. A non-significant interaction therefore means that treatment-effect modification was not demonstrated; it does not show that the SAI behaves identically across regimens. The TLS result requires the same caution. Lack of an independent association for a binary TLS variable is not inconsistent with studies linking organized lymphoid structures to antitumor immunity and treatment response (35, 42). TLS status and the SAI describe immune organization at different scales. The current data therefore support a general morphology–response association, while the separate question of whether spatial architecture predicts benefit from a specific therapy remains unanswered.

This study also has constraints that internal validation cannot resolve. It was retrospective and single-center, 176 screened patients were excluded, and treatment allocation was non-random; selection bias and residual confounding therefore remain possible. All slides were processed within one technical ecosystem, leaving uncertainty about transportability across scanners, staining practices, annotation workflows, and institutions. H&E cannot resolve immune-cell subsets or establish the molecular mechanism underlying the observed spatial patterns; the higher-dimensional studies discussed above provide biological context rather than validation of this specific index. Sparse missingness required complete-case analysis for some covariates and median imputation of one SAI component. Most importantly, the predictive estimates are internal. A convincing next study would use scanner-diverse multicenter material, prespecified processing, direct comparison with standardized stromal TIL assessment, calibration in independent populations, and an explicit test of whether the SAI adds decision-relevant value. Until such data are available, the SAI should remain a testable spatial-pathology hypothesis rather than a clinical tool.

## 5 Conclusion

In this single-center retrospective neoadjuvant TNBC cohort, an H&E-derived SAI was independently associated with pCR and provided internally validated predictive information beyond stromal TIL density. These findings justify external evaluation of interpretable spatial immune morphology metrics but do not establish clinical readiness or mechanistic immune function.

## Data Availability

The original contributions presented in the study are included in the article/**Supplementary Material**. Further inquiries can be directed to the corresponding author.

## References

[1] Bianchini G Balko JM Mayer IA Sanders ME Gianni L . Triple-negative breast cancer: challenges and opportunities of a heterogeneous disease. Nat Rev Clin Oncol. (2016) 13:674–90. doi: 10.1038/nrclinonc.2016.66 27184417 PMC5461122

[2] Foulkes WD Smith IE Reis-Filho JS . Triple-negative breast cancer. N Engl J Med. (2010) 363:1938–48. doi: 10.1056/nejmra1001389 21067385

[3] Schmid P Cortes J Dent R McArthur H Pusztai L Kummel S . Overall survival with pembrolizumab in early-stage triple-negative breast cancer. N Engl J Med. (2024) 391:1981–91. doi: 10.1056/nejmoa2409932 39282906

[4] Schmid P Cortes J Dent R Pusztai L McArthur H Kummel S . Event-free survival with pembrolizumab in early triple-negative breast cancer. N Engl J Med. (2022) 386:556–67. doi: 10.1056/nejmoa2112651 35139274

[5] Schmid P Cortes J Pusztai L McArthur H Kummel S Bergh J . Pembrolizumab for early triple-negative breast cancer. N Engl J Med. (2020) 382:810–21. doi: 10.1056/nejmoa1910549 32101663

[6] Cortazar P Zhang L Untch M Mehta K Costantino JP Wolmark N . Pathological complete response and long-term clinical benefit in breast cancer: the CTNeoBC pooled analysis. Lancet (London England). (2014) 384:164–72. doi: 10.1016/s0140-6736(13)62422-8 24529560

[7] Denkert C Loibl S Noske A Roller M Muller BM Komor M . Tumor-associated lymphocytes as an independent predictor of response to neoadjuvant chemotherapy in breast cancer. J Clin Oncol Off J Am Soc Clin Oncol. (2010) 28:105–13. doi: 10.1200/jco.2009.23.7370 19917869

[8] Denkert C von Minckwitz G Brase JC Sinn BV Gade S Kronenwett R . Tumor-infiltrating lymphocytes and response to neoadjuvant chemotherapy with or without carboplatin in human epidermal growth factor receptor 2-positive and triple-negative primary breast cancers. J Clin Oncol Off J Am Soc Clin Oncol. (2015) 33:983–91. doi: 10.1200/jco.2014.58.1967 25534375

[9] Denkert C von Minckwitz G Darb-Esfahani S Lederer B Heppner BI Weber KE . Tumour-infiltrating lymphocytes and prognosis in different subtypes of breast cancer: a pooled analysis of 3771 patients treated with neoadjuvant therapy. Lancet Oncol. (2018) 19:40–50. doi: 10.1016/s1470-2045(17)30904-x 29233559

[10] Loi S Drubay D Adams S Pruneri G Francis PA Lacroix-Triki M . Tumor-infiltrating lymphocytes and prognosis: a pooled individual patient analysis of early-stage triple-negative breast cancers. J Clin Oncol Off J Am Soc Clin Oncol. (2019) 37:559–69. doi: 10.1200/jco.18.01010 30650045 PMC7010425

[11] Ruan M Tian T Rao J Xu X Yu B Yang W . Predictive value of tumor-infiltrating lymphocytes to pathological complete response in neoadjuvant treated triple-negative breast cancers. Diagn Pathol. (2018) 13:66. doi: 10.1186/s13000-018-0743-7 30170605 PMC6119339

[12] Abuhadra N Sun R Yam C Rauch GM Ding Q Lim B . Predictive roles of baseline stromal tumor-infiltrating lymphocytes and Ki-67 in pathologic complete response in an early-stage triple-negative breast cancer prospective trial. Cancers. (2023) 15. doi: 10.3390/cancers15133275 37444385 PMC10339918

[13] Dieci MV Radosevic-Robin N Fineberg S van den Eynden G Ternes N Penault-Llorca F . Update on tumor-infiltrating lymphocytes (TILs) in breast cancer, including recommendations to assess TILs in residual disease after neoadjuvant therapy and in carcinoma in situ: a report of the International Immuno-Oncology Biomarker Working Group on Breast Cancer. Semin Cancer Biol. (2018) 52:16–25. doi: 10.1016/j.semcancer.2017.10.003 29024776

[14] Hendry S Salgado R Gevaert T Russell PA John T Thapa B . Assessing tumor-infiltrating lymphocytes in solid tumors: a practical review for pathologists and proposal for a standardized method from the International Immuno-Oncology Biomarkers Working Group: Part 2: TILs in melanoma, gastrointestinal tract carcinomas, non-small cell lung carcinoma and mesothelioma, endometrial and ovarian carcinomas, squamous cell carcinoma of the head and neck, genitourinary carcinomas, and primary brain tumors. Adv Anatomic Pathol. (2017) 24:311–35. doi: 10.1097/pap.0000000000000161 28777143 PMC5638696

[15] Salgado R Denkert C Demaria S Sirtaine N Klauschen F Pruneri G . The evaluation of tumor-infiltrating lymphocytes (TILs) in breast cancer: recommendations by an International TILs Working Group 2014. Ann Oncol Off J Eur Soc For Med Oncol. (2015) 26:259–71. doi: 10.1093/annonc/mdu450 25214542 PMC6267863

[16] Savas P Salgado R Denkert C Sotiriou C Darcy PK Smyth MJ . Clinical relevance of host immunity in breast cancer: from TILs to the clinic. Nat Rev Clin Oncol. (2016) 13:228–41. doi: 10.1038/nrclinonc.2015.215 26667975

[17] Tien TZ Lee J Lim JCT Chen XY Thike AA Tan PH . Delineating the breast cancer immune microenvironment in the era of multiplex immunohistochemistry/immunofluorescence. Histopathology. (2021) 79:139–59. doi: 10.1111/his.14328 33400265

[18] Albusayli R Graham JD Pathmanathan N Shaban M Raza SEA Minhas F . Artificial intelligence-based digital scores of stromal tumour-infiltrating lymphocytes and tumour-associated stroma predict disease-specific survival in triple-negative breast cancer. J Pathol. (2023) 260:32–42. doi: 10.1002/path.6061 36705810

[19] Fassler DJ Torre-Healy LA Gupta R Hamilton AM Kobayashi S Van Alsten SC . Spatial characterization of tumor-infiltrating lymphocytes and breast cancer progression. Cancers. (2022) 14. doi: 10.3390/cancers14092148 35565277 PMC9105398

[20] Hacking SM Karam J Singh K Gamsiz Uzun ED Brickman A Yakirevich E . Whole slide image features predict pathologic complete response and poor clinical outcomes in triple-negative breast cancer. Pathol Res Pract. (2023) 246:154476. doi: 10.1016/j.prp.2023.154476 37146413

[21] Le H Gupta R Hou L Abousamra S Fassler D Torre-Healy L . Utilizing automated breast cancer detection to identify spatial distributions of tumor-infiltrating lymphocytes in invasive breast cancer. Am J Pathol. (2020) 190:1491–504. doi: 10.1016/j.ajpath.2020.03.012 32277893 PMC7369575

[22] Baharun NB Adam A Zailani MAH Rajpoot NM Xu Q Zin RRM . Automated scoring methods for quantitative interpretation of tumour infiltrating lymphocytes (TILs) in breast cancer: a systematic review. BMC Cancer. (2024) 24:1202. doi: 10.1186/s12885-024-12962-8 39350098 PMC11440723

[23] Garcia V Elfer K Peeters DJE Ehinger A Werness B Ly A . Development of training materials for pathologists to provide machine learning validation data of tumor-infiltrating lymphocytes in breast cancer. Cancers. (2022) 14. doi: 10.3390/cancers14102467 35626070 PMC9139395

[24] Vidal JM Tsiknakis N Staaf J Bosch A Ehinger A Nimeus E . The analytical and clinical validity of AI algorithms to score TILs in TNBC: can we use different machine learning models interchangeably? EClinicalMedicine. (2024) 78:102928. doi: 10.1016/j.eclinm.2024.102928 39634035 PMC11615110

[25] Yosofvand M Khan SY Dhakal R Nejat A Moustaid-Moussa N Rahman RL . Automated detection and scoring of tumor-infiltrating lymphocytes in breast cancer histopathology slides. Cancers. (2023) 15. doi: 10.3390/cancers15143635 37509295 PMC10377197

[26] Collins GS Reitsma JB Altman DG Moons KG . Transparent reporting of a multivariable prediction model for individual prognosis or diagnosis (TRIPOD): the TRIPOD statement. BMJ (Clinical Res Ed). (2015) 350:g7594. doi: 10.1186/s12916-014-0241-z 25569120

[27] von Elm E Altman DG Egger M Pocock SJ Gotzsche PC Vandenbroucke JP . The Strengthening the Reporting of Observational Studies in Epidemiology (STROBE) statement: guidelines for reporting observational studies. Ann Internal Med. (2007) 147:573–7. doi: 10.1136/bmj.39335.541782.ad 17938396

[28] Denkert C Wienert S Poterie A Loibl S Budczies J Badve S . Standardized evaluation of tumor-infiltrating lymphocytes in breast cancer: results of the ring studies of the international immuno-oncology biomarker working group. Modern Pathol Off J United States Can Acad Pathology Inc. (2016) 29:1155–64. doi: 10.1038/modpathol.2016.109 27363491

[29] Vickers AJ Elkin EB . Decision curve analysis: a novel method for evaluating prediction models. Med Decision Making Int J Soc For Med Decision Making. (2006) 26:565–74. doi: 10.1177/0272989x06295361 17099194 PMC2577036

[30] Salgado R Denkert C Campbell C Savas P Nuciforo P Aura C . Tumor-infiltrating lymphocytes and associations with pathological complete response and event-free survival in HER2-positive early-stage breast cancer treated with lapatinib and trastuzumab: a secondary analysis of the NeoALTTO trial. JAMA Oncol. (2015) 1:448–54. doi: 10.1001/jamaoncol.2015.0830 26181252 PMC5551492

[31] Fisher TB Saini G Rekha TS Krishnamurthy J Bhattarai S Callagy G . Digital image analysis and machine learning-assisted prediction of neoadjuvant chemotherapy response in triple-negative breast cancer. Breast Cancer Res. (2024) 26:12. doi: 10.1186/s13058-023-01752-y 38238771 PMC10797728

[32] Wang XQ Danenberg E Huang CS Egle D Callari M Bermejo B . Spatial predictors of immunotherapy response in triple-negative breast cancer. Nature. (2023) 621:868–76. doi: 10.1038/s41586-023-06498-3 37674077 PMC10533410

[33] Liang H Huang J Li H He W Ao X Xie Z . Spatial proximity of CD8(+) T cells to tumor cells predicts neoadjuvant therapy efficacy in breast cancer. NPJ Breast Cancer. (2025) 11:13. doi: 10.1038/s41523-025-00728-9 39929822 PMC11811209

[34] Yan Y Lin Y Kumar T Bai S Thennavan A Li J . Ecotypes of triple-negative breast cancer in response to chemotherapy. Nature. (2026) 654:1088–97. doi: 10.1038/s41586-026-10469-9 42129561 PMC13293894

[35] Song IH Heo SH Bang WS Park HS Park IA Kim YA . Predictive value of tertiary lymphoid structures assessed by high endothelial venule counts in the neoadjuvant setting of triple-negative breast cancer. Cancer Res Treat. (2017) 49:399–407. doi: 10.4143/crt.2016.215 27488875 PMC5398384

[36] Saltz J Gupta R Hou L Kurc T Singh P Nguyen V . Spatial organization and molecular correlation of tumor-infiltrating lymphocytes using deep learning on pathology images. Cell Rep. (2018) 23:181–93. doi: 10.1016/j.celrep.2018.03.086 29617659 PMC5943714

[37] Radziuviene G Rasmusson A Augulis R Grineviciute RB Zilenaite D Laurinaviciene A . Intratumoral heterogeneity and immune response indicators to predict overall survival in a retrospective study of HER2-borderline (IHC 2+) breast cancer patients. Front Oncol. (2021) 11:774088. doi: 10.3389/fonc.2021.774088 34858854 PMC8631965

[38] Amgad M Hodge JM Elsebaie MAT Bodelon C Puvanesarajah S Gutman DA . A population-level digital histologic biomarker for enhanced prognosis of invasive breast cancer. Nat Med. (2024) 30:85–97. doi: 10.1038/s41591-023-02643-7 38012314

[39] Rijthoven MV Aswolinskiy W Tessier L Salgado R van der Laak J Ciompi F . Analysis of computational tumor-infiltrating lymphocytes in breast cancer from the results of the TIGER challenge. Nat Commun. (2026) 17:6480. doi: 10.1038/s41467-026-72956-x 42140903 PMC13377072

[40] Thagaard J Broeckx G Page DB Jahangir CA Verbandt S Kos Z . Pitfalls in machine learning-based assessment of tumor-infiltrating lymphocytes in breast cancer: a report of the International Immuno-Oncology Biomarker Working Group on Breast Cancer. J Pathol. (2023) 260:498–513. doi: 10.1002/path.6155 37608772 PMC10518802

[41] Amgad M Stovgaard ES Balslev E Thagaard J Chen W Dudgeon S . Report on computational assessment of tumor infiltrating lymphocytes from the International Immuno-Oncology Biomarker Working Group. NPJ Breast Cancer. (2020) 6:16. doi: 10.1038/s41523-020-0154-2 32411818 PMC7217824

[42] Helmink BA Reddy SM Gao J Zhang S Basar R Thakur R . B cells and tertiary lymphoid structures promote immunotherapy response. Nature. (2020) 577:549–55. doi: 10.1038/s41586-019-1922-8 31942075 PMC8762581

