# Supplementary material for: Spatial architecture of tumor-infiltrating lymphocytes adds predictive value beyond stromal TIL density for neoadjuvant response in triple-negative breast cancer
Source: Front Immunol. 2026 Sep 3;17:1879640. doi: 10.3389/fimmu.2026.1879640 (PMC13586851; doi:10.3389/fimmu.2026.1879640)
Supplement: Supplementary file 1 [file Table1.docx]

**Supplementary Materials**

***Table S1. Baseline comparison between included and excluded screened patients.***

| **Variable** | **Included, n = 236** | **Excluded, n = 176** | **P value** |
| --- | --- | --- | --- |
| Age, years | 48.6 ± 9.9 | 49.1 ± 10.1 | 0.607 |
| Body mass index, kg/m² | 24.4 ± 3.6 | 24.5 ± 3.3 | 0.920 |
| Premenopausal status | 131 (55.5%) | 94 (53.4%) | 0.746 |
| Clinical T stage |  |  | 0.521 |
| cT1 | 28 (11.9%) | 19 (10.8%) |  |
| cT2 | 140 (59.3%) | 99 (56.3%) |  |
| cT3 | 63 (26.7%) | 50 (28.4%) |  |
| cT4 | 5 (2.1%) | 8 (4.5%) |  |
| Clinical nodal status |  |  | 0.604 |
| cN0 | 115 (48.7%) | 80 (45.5%) |  |
| cN1 | 84 (35.6%) | 62 (35.2%) |  |
| cN2–3 | 37 (15.7%) | 34 (19.3%) |  |
| Histologic type |  |  | 0.864 |
| IDC | 202 (85.6%) | 148 (84.1%) |  |
| Medullary-like | 13 (5.5%) | 9 (5.1%) |  |
| Metaplastic | 4 (1.7%) | 5 (2.8%) |  |
| Other | 17 (7.2%) | 14 (8.0%) |  |
| Histologic grade 3 | 192 (81.4%) | 134 (76.1%) | 0.243 |
| Ki-67, % | 65.3 ± 15.8 | 64.8 ± 15.1 | 0.503 |
| PD-L1 CPS, available cases | 10.7 ± 8.5 | 10.6 ± 6.8 | 0.599 |
| Neoadjuvant regimen among evaluable records |  |  | 0.537 |
| Anthracycline/taxane | 104 (44.1%) | 79/160 (49.4%) |  |
| Anthracycline/taxane + carboplatin | 63 (26.7%) | 41/160 (25.6%) |  |
| Pembrolizumab + carboplatin/taxane | 69 (29.2%) | 40/160 (25.0%) |  |
| Dose reduction among evaluable records | 42 (17.8%) | 32/160 (20.0%) | 0.674 |

Values are presented as mean ± SD or n (%). Comparisons used available pretreatment data; treatment information was evaluable for 160 excluded patients.

***Table S2. Reasons for exclusion from the screened cohort.***

| **Screening step** | **n** | **% of screened cohort** | **% of excluded patients** |
| --- | --- | --- | --- |
| Initially screened TNBC patients treated with neoadjuvant therapy | 412 | 100.0 | — |
| Included in final analytic cohort | 236 | 57.3 | — |
| Excluded overall | 176 | 42.7 | 100.0 |
| Pretreatment biopsy tissue unavailable | 47 | 11.4 | 26.7 |
| Severe slide quality inadequate for digital analysis | 39 | 9.5 | 22.2 |
| Missing key clinicopathologic data | 32 | 7.8 | 18.2 |
| Non-standard neoadjuvant regimens or incomplete treatment records | 34 | 8.3 | 19.3 |
| No definitive postoperative pCR assessment at database lock | 24 | 5.8 | 13.6 |

Percentages in the final column use all excluded patients as the denominator.

***Table S3. Digital pathology workflow and quality-control parameters.***

| **Workflow domain** | **Parameter** |
| --- | --- |
| Slide source | Pretreatment core-biopsy H&E slides; one representative slide per patient |
| Section thickness | 4 μm |
| Scanner | Leica Aperio AT2 whole-slide scanner |
| Scanning magnification | 40× |
| Native resolution | 0.25 μm/pixel |
| Analysis resolution | 20× equivalent, 0.50 μm/pixel |
| File format | SVS whole-slide image |
| Software | QuPath v0.5.1; OpenSlide v4.0.0; R v4.3.2 |
| Color processing | H&E color deconvolution with Macenko-based stain normalization before batch feature extraction; no H-DAB/IHC deconvolution was used |
| Tissue detection | Optical-density threshold 0.15; minimum analyzable tissue area 1.0 mm² |
| Artifact exclusion | Necrosis, crush artifact, pen mark, fold, out-of-focus tissue, benign breast tissue, DCIS, and non-invasive structures excluded |
| Tumor annotation | Invasive tumor nests manually annotated or verified by breast pathologists blinded to pCR and treatment regimen |
| Stromal compartment | Tumor-associated stroma within invasive carcinoma; minimum stromal area 0.5 mm² |
| Nuclei segmentation | StarDist 2D; probability threshold 0.50; non-maximum-suppression overlap 0.40 |
| Lymphocyte classifier | Locked random-forest classifier trained on 2,800 pathologist-labeled nuclei from 30 representative slides |
| Held-out classifier performance | Accuracy 0.89; precision 0.87; recall 0.85; F1 score 0.86 |
| Close interaction threshold | 20 μm from lymphocyte centroid to nearest tumor boundary |
| Local clustering radius | 50 μm lymphocyte-neighborhood radius |
| Edge band | 0–50 μm tumor–stroma interface band |
| Quality-control categories | Pass, minor artifact, segmentation review |
| Analytic cohort quality result | Pass 194/236; minor artifact 29/236; segmentation review 13/236 |
| Severe quality exclusion rule | Severe blur/fold/crush/necrosis affecting >30% of analyzable tumor–stroma interface or failed tumor/stroma annotation |

***Table S4. Spatial descriptors, primary SAI components, directionality, and cohort-level standardization parameters.***

| **Descriptor** | **Role** | **Direction in SAI** | **Reverse-coded** | **Imputation median** | **Reference mean** | **Reference SD** | **Rationale** |
| --- | --- | --- | --- | --- | --- | --- | --- |
| Pathologist-read stromal TIL density | Density descriptor; not in primary SAI | Not applicable | No | — | — | — | Global stromal immune abundance |
| Digital TIL area | Density descriptor; not in primary SAI | Not applicable | No | — | — | — | Digitally measured stromal lymphocyte area |
| Close interaction ratio within 20 μm | Primary SAI component | Positive | No | 0.469 | 0.467 | 0.232 | Higher values indicate greater tumor proximity |
| Lymphocyte cluster index | Primary SAI component | Positive | No | 1.270 | 1.350 | 0.545 | Higher values indicate greater local aggregation |
| Mean lymphocyte–tumor distance | Primary SAI component | Negative | Yes | 41.250 | 41.477 | 9.217 | Longer distance indicates less tumor-proximal organization |
| Edge enrichment score | Primary SAI component | Negative | Yes | 66.350 | 63.561 | 21.802 | Greater edge restriction indicates margin-limited accumulation |

Primary formula: SAIraw = [z(close interaction ratio) + z(cluster index) − z(mean lymphocyte–tumor distance) − z(edge enrichment)]/4. For the primary regression analysis, the cohort-level parameters shown above were used and SAIraw was then restandardized to a mean of 0 and an SD of 1. During nested cross-validation, imputation and standardization parameters were estimated within each training fold and applied unchanged to the held-out fold.

***Table S5. Operational criteria and quantitative summary of the four representative spatial phenotypes.***

| **Phenotype** | **Dominant operational pattern** | **N** | **pCR, n (%)** | **Median stromal TILs (%)** | **Median digital TIL area (%)** | **Median distance (μm)** | **Median close interaction ratio** | **Median cluster index** | **Median edge enrichment** | **Median SAI** |
| --- | --- | --- | --- | --- | --- | --- | --- | --- | --- | --- |
| Immune-excluded | Low inflammation and low proximity; lymphocytes predominantly remote from tumor nests, usually with high distance and high edge enrichment. | 62 | 22 (35.5%) | 11.0 | 7.2 | 48.4 | 0.213 | 0.94 | 84.8 | -0.87 |
| Margin-restricted | Lymphocytes concentrated in the 0–50 μm interface band, with high edge enrichment and low-to-moderate close interaction. | 67 | 30 (44.8%) | 35.0 | 34.6 | 44.5 | 0.346 | 1.06 | 76.3 | -0.46 |
| Inflamed-dispersed | Moderate-to-high inflammation distributed through tumor-associated stroma, with higher proximity and lower edge restriction but without dense perinest clustering. | 65 | 40 (61.5%) | 32.0 | 36.4 | 36.9 | 0.629 | 1.42 | 51.1 | 0.44 |
| Inflamed-proximal | High inflammation with abundant close interaction and clustering adjacent to tumor nests, low distance, and low edge restriction. | 42 | 30 (71.4%) | 68.0 | 71.1 | 29.1 | 0.761 | 2.00 | 33.5 | 1.29 |

Phenotypes were assigned according to the dominant pattern across annotated invasive tumor and associated stroma. When patterns coexisted, the phenotype occupying the largest analyzable area was assigned after review. All 236 cases were classifiable.

***Table S6. Reproducibility of pathological and spatial measurements.***

| **Variable** | **Statistic** | **Estimate** | **95% CI** | **Additional agreement metric** |
| --- | --- | --- | --- | --- |
| Pathologist-read stromal TILs, % | ICC(2,1) | 0.94 | 0.91–0.96 | Mean absolute difference 7.7% |
| Digital TIL area, % | ICC(2,1) | 0.96 | 0.93–0.97 | Mean absolute difference 7.0% |
| Mean lymphocyte–tumor distance, μm | ICC(2,1) | 0.91 | 0.87–0.93 | Mean absolute difference 3.7 μm |
| Close interaction ratio within 20 μm | ICC(2,1) | 0.97 | 0.95–0.98 | Mean absolute difference 0.055 |
| Lymphocyte cluster index | ICC(2,1) | 0.90 | 0.85–0.94 | Mean absolute difference 0.17 |
| Edge enrichment score | ICC(2,1) | 0.91 | 0.87–0.94 | Mean absolute difference 8.3 |
| Spatial architecture index | ICC(2,1) | 0.93 | 0.89–0.95 | Mean absolute difference 0.35 SD units |
| TLS presence | Cohen’s κ | 0.83 | 0.66–0.96 | Agreement 91.7% |
| Spatial phenotype, four-category | Unweighted κ | 0.79 | 0.66–0.91 | Agreement 85.0% |
| Image-quality category | Unweighted κ | 0.75 | 0.46–0.94 | Agreement 93.3% |
| pCR re-abstraction | Cohen’s κ | 0.97 | 0.90–1.00 | Agreement 98.3% |

The reread subset included 60 cases stratified 1:1 by pCR status. Two breast pathologists were blinded to pCR status, treatment regimen, and each other’s assessments. Continuous variables were evaluated with a two-way random-effects, absolute-agreement, single-measure ICC [ICC(2,1)].

***Table S7. Missing-data profile and analysis sample sizes.***

Panel A. Variables with non-zero missingness.

| **Variable** | **Missing n** | **Total n** | **Missing %** |
| --- | --- | --- | --- |
| PD-L1 CPS | 14 | 236 | 5.9% |
| Close interaction ratio within 20 μm | 7 | 236 | 3.0% |
| Body mass index | 6 | 236 | 2.5% |
| Digital TIL area | 6 | 236 | 2.5% |
| Ki-67 | 5 | 236 | 2.1% |
| pCR outcome | 0 | 236 | 0.0% |

Panel B. Analysis denominators.

| **Analysis** | **N** |
| --- | --- |
| Total analytic cohort | 236 |
| Main multivariable model | 231 |
| Complete-case four-component SAI sensitivity analysis | 224 |
| PD-L1/TLS-extended complete-case dataset | 211 |

***Table S8. Sensitivity analyses of alternative spatial architecture index constructions.***

| **Analysis** | **N** | **Odds ratio (95% CI)** | **P value** |
| --- | --- | --- | --- |
| Primary 4-component equal-weight SAI with component median imputation | 231 | 2.79 (1.79 to 4.35) | <0.001 |
| Complete-case 4-component equal-weight SAI | 224 | 2.74 (1.76 to 4.28) | <0.001 |
| Leave-one-component-out SAI: excluding close interaction ratio | 231 | 2.66 (1.72 to 4.12) | <0.001 |
| Leave-one-component-out SAI: excluding lymphocyte cluster index | 231 | 2.70 (1.76 to 4.14) | <0.001 |
| Leave-one-component-out SAI: excluding mean lymphocyte–tumor distance, μm | 231 | 2.62 (1.70 to 4.04) | <0.001 |
| Leave-one-component-out SAI: excluding edge enrichment score | 231 | 2.70 (1.74 to 4.20) | <0.001 |
| Rank-based 4-component SAI | 231 | 2.75 (1.79 to 4.21) | <0.001 |
| Principal-component-derived 4-component SAI | 231 | 2.79 (1.80 to 4.34) | <0.001 |
| Exploratory six-descriptor SAI including density descriptors | 231 | 1.99 (1.42 to 2.79) | <0.001 |

All models included the primary clinicopathologic covariates. Four-component SAI analyses additionally adjusted for stromal TIL density. Because the exploratory six-descriptor index incorporated density measures, stromal TIL density was not included as a separate covariate in that model.

***Table S9. Treatment-regimen, temporal, interaction, and propensity-score sensitivity analyses.***

Panel A. Subgroup, calendar-year, treatment-period, and interaction analyses.

| **Analysis** | **N** | **Odds ratio (95% CI)** | **P value** |
| --- | --- | --- | --- |
| Chemotherapy-only subgroup | 165 | 2.48 (1.52 to 4.05) | <0.001 |
| Pembrolizumab-containing subgroup | 66 | 5.23 (1.75 to 15.66) | 0.003 |
| Main model additionally adjusted for diagnosis year | 231 | 2.82 (1.81 to 4.41) | <0.001 |
| Main model additionally adjusted for treatment period | 231 | 2.80 (1.80 to 4.36) | <0.001 |
| SAI × pembrolizumab-containing treatment interaction | 231 | 1.75 (0.71 to 4.32) | 0.222 |

Panel B. Propensity-score analyses for receipt of pembrolizumab-containing therapy.

| **Analysis** | **N** | **Odds ratio (95% CI)** | **P value** |
| --- | --- | --- | --- |
| Propensity-score logit adjusted outcome model | 236 | 2.81 (1.88 to 4.19) | <0.001 |
| IPTW weighted outcome model | 236 | 2.61 (1.54 to 4.43) | <0.001 |
| Overlap weighted outcome model | 236 | 3.14 (1.69 to 5.85) | <0.001 |

The propensity model included age, clinical T stage, clinical nodal status, grade, Ki-67, stromal TIL density, SAI, PD-L1 CPS, TLS, and diagnosis year. After overlap weighting, the largest absolute standardized mean difference was 0.064.

***Table S10. Correlation, collinearity, and stromal TIL attenuation diagnostics.***

Panel A. Correlation between the SAI and stromal TIL density.

| **Comparison** | **Spearman ρ** | **P value** | **N** |
| --- | --- | --- | --- |
| SAI vs stromal TIL density | 0.589 | <0.001 | 231 |

Panel B. Adjusted variance inflation factors from the main model.

| **Variable** | **Adjusted VIF** |
| --- | --- |
| Age, per year | 1.02 |
| Clinical T stage, cT3–4 vs cT1–2 | 1.05 |
| Clinical nodal status, cN+ vs cN0 | 1.04 |
| Histologic grade, 3 vs 2 | 1.13 |
| Ki-67, per 10% | 1.14 |
| Neoadjuvant regimen | 1.02 |
| Stromal TIL density, per 10% | 1.28 |
| Spatial architecture index, per 1 SD | 1.32 |

Panel C. Stromal TIL coefficient before and after SAI inclusion.

| **Analysis** | **Odds ratio (95% CI)** | **P value** |
| --- | --- | --- |
| Clinical covariates + stromal TIL density | 1.12 (0.99 to 1.27) | 0.077 |
| Clinical covariates + stromal TIL density + SAI | 0.88 (0.74 to 1.04) | 0.123 |

***Table S11. Apparent and bootstrap optimism-corrected performance of sequential logistic models.***

| **Model** | **Performance type** | **N** | **Bootstrap resamples** | **AUROC** | **AUPRC** | **Brier** | **Calibration intercept** | **Calibration slope** |
| --- | --- | --- | --- | --- | --- | --- | --- | --- |
| Clinical model | Apparent | 231 | 500 | 0.730 | 0.723 | 0.211 | -0.000 | 1.000 |
| Clinical model | Bootstrap optimism-corrected | 231 | 500 | 0.698 | 0.689 | 0.226 | -0.001 | 0.840 |
| Clinical + density | Apparent | 231 | 500 | 0.740 | 0.722 | 0.208 | -0.000 | 1.000 |
| Clinical + density | Bootstrap optimism-corrected | 231 | 500 | 0.705 | 0.686 | 0.224 | 0.001 | 0.825 |
| Clinical + SAI | Apparent | 231 | 500 | 0.794 | 0.793 | 0.187 | 0.000 | 1.000 |
| Clinical + SAI | Bootstrap optimism-corrected | 231 | 500 | 0.764 | 0.764 | 0.203 | -0.000 | 0.855 |
| Clinical + density + SAI | Apparent | 231 | 500 | 0.797 | 0.803 | 0.185 | 0.000 | 1.000 |
| Clinical + density + SAI | Bootstrap optimism-corrected | 231 | 500 | 0.765 | 0.771 | 0.203 | 0.001 | 0.839 |

***Table S12. Sensitivity multivariable logistic regression additionally adjusted for tertiary lymphoid structures.***

| **Variable** | **Odds ratio (95% CI)** | **P value** |
| --- | --- | --- |
| Clinical T stage, cT3-4 vs cT1-2 | 0.46 (0.22 to 0.93) | 0.030 |
| Clinical nodal status, cN+ vs cN0 | 0.72 (0.39 to 1.35) | 0.307 |
| Histologic grade, 3 vs 2 | 2.57 (1.05 to 6.28) | 0.039 |
| Anthracycline/taxane + carboplatin vs anthracycline/taxane | 1.44 (0.69 to 2.99) | 0.327 |
| Pembrolizumab + carboplatin/taxane vs anthracycline/taxane | 3.13 (1.47 to 6.64) | 0.003 |
| TLS present, yes vs no | 0.96 (0.49 to 1.87) | 0.906 |
| Age (per year) | 1.02 (0.98 to 1.05) | 0.311 |
| Ki-67 (per 10%) | 1.18 (0.96 to 1.46) | 0.122 |
| Pathologist-read stromal TILs (per 10%) | 0.88 (0.74 to 1.04) | 0.124 |
| Spatial architecture index (per 1 SD) | 2.81 (1.78 to 4.45) | <0.001 |

The analytic sample included 231 patients with complete clinicopathologic covariates. Ki-67 and stromal TIL density were modeled per 10% increase, and the SAI per 1 SD. Reference categories were cT1–2, cN0, grade 2, the anthracycline/taxane regimen, and absence of TLS.

***Table S13. Spearman correlation matrix of density and spatial TIL features.***

| **Variable** | **Stromal TILs** | **Digital TIL area** | **Lymphocyte–tumor distance** | **Close interaction ratio** | **Cluster index** | **Edge enrichment** | **SAI** |
| --- | --- | --- | --- | --- | --- | --- | --- |
| Stromal TILs | 1.00 |  |  |  |  |  |  |
| Digital TIL area | 0.93 | 1.00 |  |  |  |  |  |
| Lymphocyte–tumor distance | -0.54 | -0.61 | 1.00 |  |  |  |  |
| Close interaction ratio | 0.59 | 0.64 | -0.74 | 1.00 |  |  |  |
| Cluster index | 0.51 | 0.57 | -0.64 | 0.71 | 1.00 |  |  |
| Edge enrichment | -0.51 | -0.57 | 0.72 | -0.80 | -0.70 | 1.00 |  |
| Spatial architecture index | 0.60 | 0.67 | -0.90 | 0.91 | 0.80 | -0.89 | 1.00 |

Coefficients were calculated using complete cases. Only the lower triangle is shown.

***Table S14. Repeated nested cross-validation performance across all feature sets and algorithms.***

| **Feature set** | **Model** | **AUROC, mean ± SD** | **AUROC empirical interval** | **AUPRC, mean ± SD** | **Brier, mean ± SD** | **Resamples** |
| --- | --- | --- | --- | --- | --- | --- |
| FS1: Clinical | Ridge regression | 0.689 ± 0.067 | 0.545 to 0.799 | 0.705 ± 0.070 | 0.239 ± 0.011 | 50 |
| FS1: Clinical | Random forest | 0.687 ± 0.081 | 0.501 to 0.828 | 0.715 ± 0.072 | 0.227 ± 0.016 | 50 |
| FS1: Clinical | Logistic regression | 0.684 ± 0.063 | 0.557 to 0.784 | 0.697 ± 0.067 | 0.229 ± 0.022 | 50 |
| FS1: Clinical | Elastic Net | 0.681 ± 0.067 | 0.548 to 0.799 | 0.693 ± 0.072 | 0.231 ± 0.016 | 50 |
| FS1: Clinical | XGBoost | 0.679 ± 0.074 | 0.536 to 0.798 | 0.708 ± 0.073 | 0.238 ± 0.017 | 50 |
| FS1: Clinical | Linear SVM | 0.675 ± 0.068 | 0.538 to 0.786 | 0.675 ± 0.075 | 0.226 ± 0.014 | 50 |
| FS2: Clinical + TIL density | Ridge regression | 0.702 ± 0.067 | 0.580 to 0.802 | 0.711 ± 0.070 | 0.236 ± 0.012 | 50 |
| FS2: Clinical + TIL density | Linear SVM | 0.696 ± 0.063 | 0.567 to 0.820 | 0.702 ± 0.070 | 0.228 ± 0.016 | 50 |
| FS2: Clinical + TIL density | Logistic regression | 0.694 ± 0.065 | 0.576 to 0.804 | 0.703 ± 0.071 | 0.226 ± 0.025 | 50 |
| FS2: Clinical + TIL density | Random forest | 0.694 ± 0.073 | 0.564 to 0.833 | 0.706 ± 0.071 | 0.227 ± 0.016 | 50 |
| FS2: Clinical + TIL density | Elastic Net | 0.691 ± 0.067 | 0.555 to 0.798 | 0.700 ± 0.068 | 0.228 ± 0.016 | 50 |
| FS2: Clinical + TIL density | XGBoost | 0.676 ± 0.073 | 0.516 to 0.790 | 0.696 ± 0.070 | 0.239 ± 0.019 | 50 |
| FS3: Clinical + TIL density + SAI | Logistic regression | 0.752 ± 0.066 | 0.629 to 0.877 | 0.769 ± 0.075 | 0.207 ± 0.030 | 50 |
| FS3: Clinical + TIL density + SAI | Elastic Net | 0.751 ± 0.065 | 0.631 to 0.875 | 0.766 ± 0.072 | 0.207 ± 0.024 | 50 |
| FS3: Clinical + TIL density + SAI | Ridge regression | 0.751 ± 0.064 | 0.637 to 0.867 | 0.767 ± 0.071 | 0.214 ± 0.021 | 50 |
| FS3: Clinical + TIL density + SAI | Linear SVM | 0.749 ± 0.063 | 0.630 to 0.869 | 0.767 ± 0.068 | 0.208 ± 0.020 | 50 |
| FS3: Clinical + TIL density + SAI | Random forest | 0.746 ± 0.065 | 0.625 to 0.876 | 0.767 ± 0.070 | 0.212 ± 0.016 | 50 |
| FS3: Clinical + TIL density + SAI | XGBoost | 0.730 ± 0.072 | 0.599 to 0.854 | 0.760 ± 0.077 | 0.226 ± 0.023 | 50 |

***Table S15. Net benefit of full-feature models at selected threshold probabilities.***

| **Threshold** | **Treat all** | **Treat none** | **Logistic regression** | **Elastic Net** | **Ridge regression** | **Linear SVM** | **Random forest** | **XGBoost** |
| --- | --- | --- | --- | --- | --- | --- | --- | --- |
| 0.10 | 0.463 | 0.000 | 0.445 | 0.463 | 0.463 | 0.463 | 0.463 | 0.463 |
| 0.20 | 0.396 | 0.000 | 0.386 | 0.388 | 0.395 | 0.388 | 0.396 | 0.396 |
| 0.30 | 0.310 | 0.000 | 0.325 | 0.323 | 0.322 | 0.321 | 0.310 | 0.318 |
| 0.40 | 0.195 | 0.000 | 0.260 | 0.273 | 0.247 | 0.263 | 0.247 | 0.246 |

Net-benefit curves were closely grouped across models. Values are shown at selected threshold probabilities to quantify the limited differences among algorithms.

**Supplementary Figures**

***Figure S1. Study flowchart of patient selection and analytic cohort derivation.***

The flowchart shows 412 screened patients, the reasons for exclusion, the final analytic cohort of 236 patients, image-quality categories, and pathologic response groups.

***Figure S2. Distribution of representative spatial morphology metrics according to pathologic response.***

Violin plots with embedded boxplots and individual observations compare edge enrichment, stromal TIL density, and standardized SAI between the non-pCR and pCR groups. P values were calculated using two-sided Wilcoxon rank-sum tests.

***Figure S3. Fold-level distributions of model performance for the full feature set.***

Distributions across 50 outer resamples are shown for (A) AUROC, (B) AUPRC, and (C) Brier score.

***Figure S4. Stepwise performance across predefined feature sets.***

Mean ± SD performance across FS1 (clinical variables), FS2 (clinical variables + stromal TIL density), and FS3 (clinical variables + stromal TIL density + SAI) is shown for (A) AUROC, (B) AUPRC, and (C) Brier score.
